# Bio-Based Solvents for Green Extraction of Lipids from Oleaginous Yeast Biomass for Sustainable Aviation Biofuel

**DOI:** 10.3390/molecules21020196

**Published:** 2016-02-06

**Authors:** Cassandra Breil, Alice Meullemiestre, Maryline Vian, Farid Chemat

**Affiliations:** Laboratoire GREEN, Université d’Avignon, Vaucluse, INRA, UMR 408, GREEN Extraction Team, F-84000 Avignon, France; cassandra.breil@alumni.univ-avignon.fr (C.B.); alice.meullemiestre@univ-avignon.fr (A.M.); farid.chemat@univ-avignon.fr (F.C.)

**Keywords:** yeast, lipids, COSMO-RS, Hansen, bio-based solvents

## Abstract

Lipid-based oleaginous microorganisms are potential candidates and resources for the sustainable production of biofuels. This study was designed to evaluate the performance of several alternative bio-based solvents for extracting lipids from yeasts. We used experimental design and simulation with Hansen solubility simulations and the conductor-like screening model for realistic solvation (COSMO-RS) to simulate the solubilization of lipids in each of these solvents. Lipid extracts were analyzed by high performance thin-layer chromatography (HPTLC) to obtain the distribution of lipids classes and gas chromatography coupled with a flame ionization detector (GC/FID) to obtain fatty acid profiles. Our aim was to correlate simulation with experimentation for extraction and solvation of lipids with bio-based solvents in order to make a preliminary evaluation for the replacement of hexane to extract lipids from microorganisms. Differences between theory and practice were noted for several solvents, such as CPME, MeTHF and ethyl acetate, which appeared to be good candidates to replace hexane.

## 1. Introduction

Like every other mode of transport, aviation has to respond to the challenges raised by climate change. The combustion of fuels contributes to the greenhouse effect due to carbon emission. In the face of petroleum resource depletion and concern about the environment, the development of renewable, sustainable and efficient energy sources is being strongly encouraged.

Lipid-based biomass is being increasingly considered as an alternative raw material for vehicle fuels. Lipid-based biomasses derived from edible oil crops, such as rapeseed, soybean and maize, form the first generation feedstock, because of their ready availability. However, the use of first generation biofuels is controversial due to their competition with food. The development of non-food oil plants has thus been proposed to provide feedstock, such as Camelina and jatropha. A number of test flights have already been conducted with jatropha or Camelina biofuel blended with conventional kerosene. Lipids can also be produced by oleaginous microorganisms, such as microalgae, yeast, fungi or bacteria. Microbial lipids are potential candidates and resources for the sustainable production of biofuels and value-added bioproducts and offer an alternative route to replace petroleum-based hydrocarbons and chemicals. For example, microalgae have been recognized as potentially good sources of biofuel because they synthesize and accumulate large quantities of neutral lipids (20%–50% dry weight of biomass) and grow fast [1].

Among microorganisms, oleaginous yeasts have been reported as good producers of lipids, mostly TAGs. They can accumulate more than 20% of their dry cell mass as lipids [2] converting carbon sources contained in various substrates. Under specific culture conditions, some species can accumulate up to 70% of their dry cell weight as lipids [3]. Moreover, microbial oil exhibits a significant proportion of various fatty acids and thus offers good potential as possible sources for biofuels.

*Yarrowia lipolytica* is recognized as being a GRAS organism (generally recognized as safe). It can assimilate a wide variety of carbon substrates and metabolizes hydrocarbons, such as [4], and lipids, such as fatty acids (mainly triglycerides) [5].

Scientific and industrial research laboratories face the challenge of finding an appropriate extraction method with minimum energy consumption and green solvents. Today, the most common technique for extraction of lipids from microorganisms is generally solvent extraction, using petroleum solvents, such as hexane or mixtures of hydrocarbons [6]. From the point of view of environmental protection and the development of green chemistry, these flammable and toxic petroleum solvents [7] will have to be replaced in the future by bio-based solvents (or “bio-solvents”) [8]. These bio-based solvents are mostly produced from agricultural sources and are potential candidates to replace petroleum-derived solvents.

**Figure 1:**
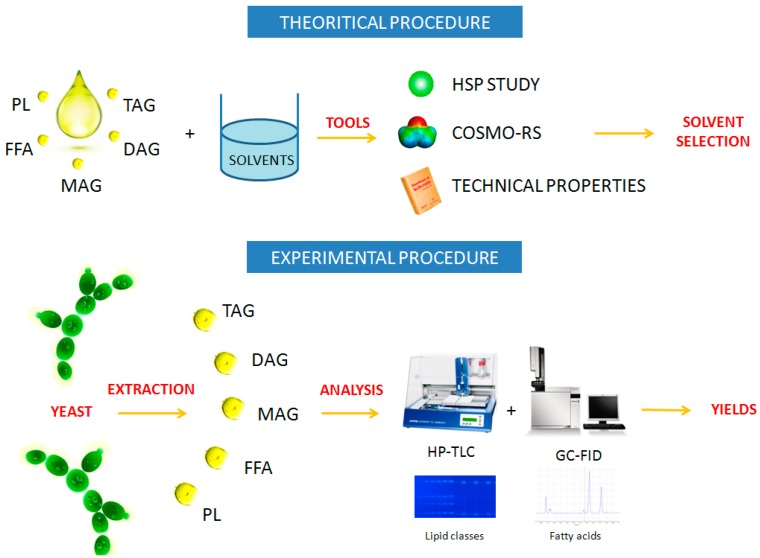
The aim is to compare both scientific approaches (theoretical procedure *vs*. experimental procedure) and to show the global procedure.

The present work combines an experimental study with two theoretical approaches (Figure 1). The experimental part is based on the extraction of oil from *Yarrowia lipolytica* IFP29. The oil obtained was analyzed by high performance thin-layer chromatography (HPTLC) to obtain lipid classes and gas chromatography coupled with a flame ionization detector (GC/FID) for fatty acid profiles.

The first theoretical approach used Hansen solubility parameters, which evaluate the interactions between solvents and solute (FFAs, DAGs, TAGs and PLs) present in the matrix. This was extended by a more precise theoretical approach: the conductor-like screening model for realistic solvation (COSMO-RS). It simulates the relative solubility of the solute in the selected solvent, here 2-methyltetrahydrofuran (MeTHF), cyclopentyl methyl ether (CPME), isopropanol (IPA), ethanol (EtOH), ethyl acetate (EtOAc), ethyl lactate, dimethyl carbonate (DMC), p-cymene, *d*-Limonene, α-pinene and hexane.

We went on to compare the experiment and theoretical parts and make the first inventory of potential bio-based solvents that could replace hexane for the extraction of yeast oil.

## 2. Results and Discussion

For all of the experiments, biomass (*Yarrowia lipolytica* IFP29) was extracted with the different types of solvent (hexane, isopropanol, ethanol, ethyl acetate, ethyl lactate, DMC, p-cymene, α-pinene, *d*-Limonene, MeTHF and CPME) for 30 min by maceration. The extracts are centrifuged and evaporated. The oil obtained was analyzed by high performance thin-layer chromatography (HPTLC) to obtain lipid classes and by gas chromatography coupled with a flame ionization detector (GC/FID) after transmethylation to obtain fatty acid profiles.

Then, all of the alternative solvents were compared with hexane, the reference for the extraction of fat and oil in research laboratories and in industry [9].

Our experimental results were compared to those of the simulation approach (software Hansen and COSMO-RS) to evaluate the possible replacement of hexane by alternative solvents for the extraction of microbial oil.

### 2.1. Qualitative and Quantitative Comparison of Extracts

#### 2.1.1. Gas Chromatography

**Figure 2:**
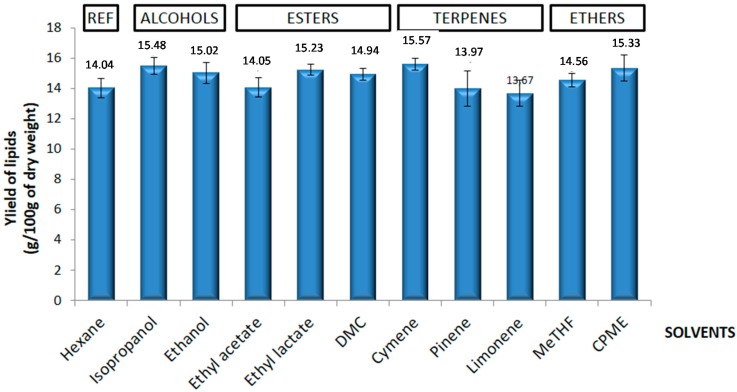
Comparison of yields obtained by several solvents to determinate the best solvent for the extraction of yeast oil.

As shown in Figure 2, lipid yields were around 15%, with no significant difference between hexane and the other solvents.

**Figure 3:**
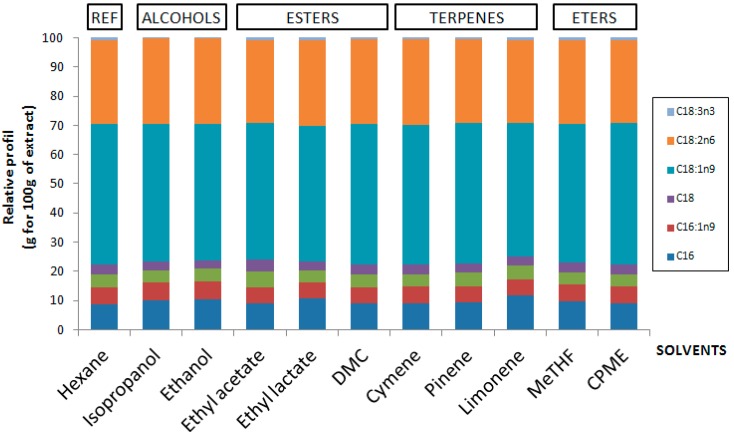
Comparison of the distribution of fatty acids by GC-FID to determinate if there exists a selectivity during extraction.

Fatty acid profiles (Figure 3) show MeTHF, CPME, isopropanol, ethanol, ethyl acetate, ethyl lactate, DMC, p-cymene, *d*-Limonene and α-pinene to be similar to hexane in terms of selectivity. Profiles show that oleic acid (C18:1), linoleic acid (C18:2n6) and palmitic acid (C16:0) were mainly present in microbial oil and represented at least 90% of the total extract (respectively about 50% of C18:1, 30% of C18:2n6 and 10% of C16:0). Palmitoleic acid (C16:1) and stearic acids were in minor amounts. The detailed composition for each extract is reported in Table 1.

**Table 1:** Crude lipids, distribution of lipid classes and fatty acid compositions of extracts obtained by extractions with different solvents (PUFAs, polyunsaturated fatty acids; MUFAs, monounsaturated fatty acids; SFAs, saturated fatty acids).

	Solvents										
	Conventional Solvents	Alternative Solvents									
	Hexane	Isopropanol	Ethanol	Ethyl acetate	Ethyl lactate	DMC	Cymene	Pinene	Limonene	MeTHF	CPME
**Lipids Yieds**	14.03 ± 0.67	15.48 ± 0.57	15.02 ± 0.71	12.63 ± 0.64	15.53 ± 0.37	14.93 ± 0.39	15.57 ± 0.39	13.96 ± 1.16	13.67 ± 0.83	15.94 ± 0.44	15.33 ± 0.87
**Lipid classes composition (%)**											
FFA: Free fatty acid	13.30 ± 0.95	17.41 ± 2.75	9.07 ± 0.40	15.06 ± 0.49	21.97 ± 2.15	24.56 ± 1.03	21.18 ± 2.13	20.78 ± 1.07	12.97 ± 0.18	14.75 ± 1.28	16.90 ± 1.48
TAG: Triacylglycerol	71.70 ± 0.81	54.71 ± 1.73	60.08 ± 1.19	59.33 ± 1.12	61.92 ± 2.51	64.56 ± 1.85	62.17 ± 1.49	64.17 ± 0.98	76.10 ± 2.58	69.34 ± 2.00	59.88 ± 3.89
DAG: Diacylglycerol	14.99 ± 1.01	24.41 ± 0.84	25.63 ± 0.48	24.34 ± 1.18	12.96 ± 0.44	19.59 ± 1.90	16.64 ± 2.20	15.04 ± 1.45	10.93 ± 2.40	12.81 ± 1.06	22.27 ± 1.93
PE: Phosphatdyléthanolamine	-	1.77 ± 0.04	3.11 ± 0.15	1.25 ± 0.34	3.11 ± 0.19	0.93 ± 0.10	-	-	-	-	-
PC: Phosphatydylcholine	-	1.69 ± 0.21	2.08 ± 0.15	-	-	-	-	-	-	-	-
**Fatty acids composition (%)**											
*Satured*											
C16	9.15 ± 0.13	10.37 ± 0.04	10.95 ± 0.13	9.461 ± 0.14	11.041 ± 0.24	9.28 ± 0.85	9.34 ± 0.41	9.70 ± 0.58	12.34 ± 0.89	10.13 ± 0.75	9.34 ± 0.26
C18	3.37 ± 0.15	3.12 ± 0.04	3.02 ± 0.06	4.52 ± 0.32	2.92 ± 0.55	3.31 ± 0.03	3.28 ± 0.09	3.25 ± 0.29	3.27 ± 0.23	3.39 ± 0.20	3.41 ± 0.04
*Mono-unsatured*											
C16:1n9	5.92 ± 0.03	6.37 ± 0.03	6.38 ± 0.02	5.92 ± 0.08	5.86 ± 0.77	5.98 ± 0.03	6.01 ± 0.07	5.96 ± 0.1	5.57 ± 0.04	6.12 ± 0.04	6.00 ± 0.05
C18:1n9	50.71 ± 0.07	49.24 ± 0.33	48.6 ± 0.15	49.39 ± 0.71	48.73 ± 0.24	50.46 ± 0.28	50.22 ± 0.26	50.28 ± 0.11	48.29 ± 0.11	49.63 ± 0.18	50.64 ± 0.05
*Poly-unsatured*											
C18:2n6	30.02 ± 0.19	30.68 ± 0.03	30.7 ± 0.04	29.95 ± 0.15	30.6 ± 0.58	30.45 ± 0.1	30.45 ± 0.08	30.1 ± 0.35	29.7 ± 0.11	29.93 ± 0.11	29.75 ± 0.35
C18:3n3	0.81 ± 0.01	0.19 ± 0.17	0.33 ± 0.37	0.75 ± 0.02	0.84 ± 0.39	0.5 ± 0.27	0.68 ± 0.11	0.70 ± 0.02	0.82 ± 0.02	0.77 ± 0.15	0.85 ± 0.25
∑ SFAs	12.53	13.51	13.98	13.98	13.97	12.61	12.64	12.97	15.62	13.54	12.76
∑ MUFAs	56.64	55.62	54.99	55.32	54.60	56.44	56.24	56.24	53.86	55.76	56.64
∑ PUFAs	30.83	30.87	31.03	30.70	31.44	30.95	31.13	30.80	30.52	30.70	30.60

#### 2.1.2. High Performance Thin-Layer Chromatography

**Figure 4:**
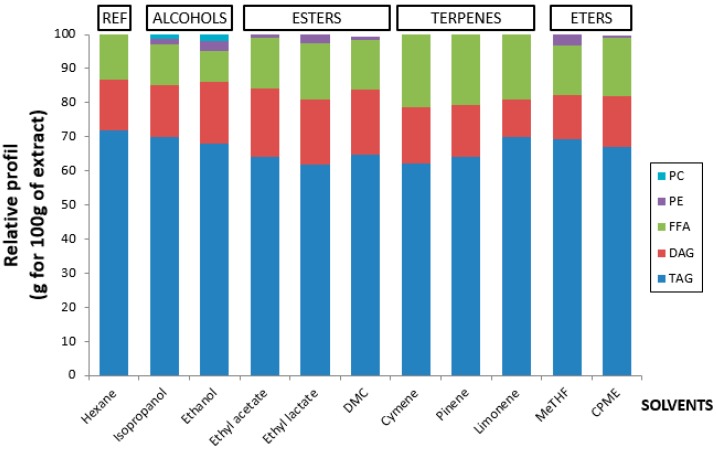
Comparison of lipid classes (TAG, DAG, MAG, FFA and PL) by HPTLC.

Concerning lipid classes (Figure 4), 60%–70% of total lipids in all of the solvent extracts were triglycerides (TAGs), about 10%–20% diglycerides (DAGs) and 10%–20% free fatty acids (FFAs). Selectivity is noted for compounds such as phospholipids; for example, phosphatidylethanolamine (PE), which represents less than 4% of the extract for isopropanol, ethanol, ethyl acetate, DMC, MeTHF and CPME. Phosphatidylcholine (PC) was extracted only by isopropanol and ethanol, at less than 2%. The presence of these phospholipids is due to the high polarity of the solvents used, such as ethanol and isopropanol. The detailed composition for each extract is reported in Table 1.

### 2.2. Modelling and Prediction Study

We evaluated the potential of alternative solvents for the extraction of oil from *Yarrowia lipolytica* IFP29. This study was conducted with predictive software COSMO-RS (conductor-like screening model for realistic solvation) and Hansen solubility parameters. The theoretical predictions were compared to the practical results for fatty acid profiles and lipid classes. 

**Figure 5:**
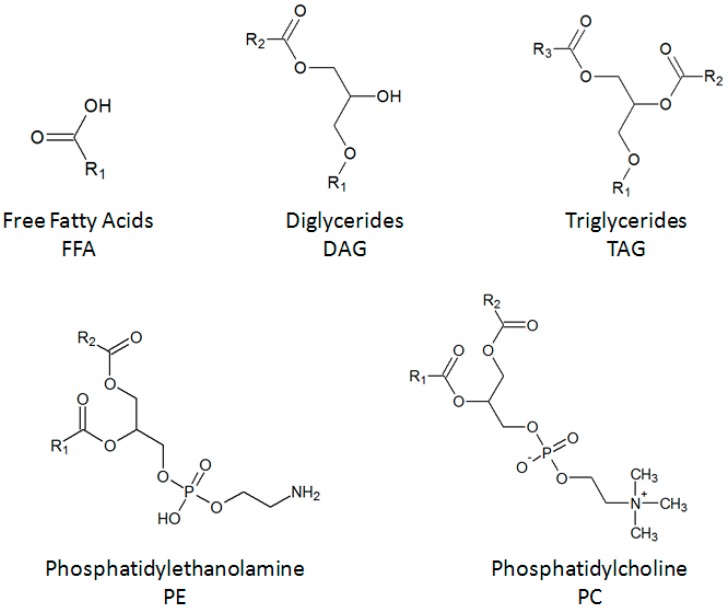
Structure of major lipids (FFA, DAG, TAG, PE, PC) found in yeast (*Yarrowia lipolytica*).

To conduct a predictive study, we have to know the most abundant molecules (Figure 5) in oil from *Yarrowia Lipolytica* IFP29. HPTLC results show that the main classes of lipids present in the oil were TAG, DAG and FFA. GC analysis showed that these lipids were composed of long carbon chains, such as C16, C18:1n9 and C18:2n6. The main components were modelled by the ChemSketch software and used for the predictive study.

#### 2.2.1. Hansen Solubility Parameters

Based on thermodynamic principles of molecules, Hansen evaluates the solubility potential of lipids (Table S1 in Supplementary Materials) (TAG, DAG, FFA) in the selected bio-based solvents (hexane, isopropanol, ethanol, ethyl acetate, ethyl lactate, DMC, p-cymene, α-pinene, *d*-Limonene, MeTHF and CPME). The ability of the solvent to solubilize the solute is represented by the relative energy difference (RED). In Table 2, p-cymene, *d*-Limonene, α-pinene, MeTHF and CPME have RED < 1, which means that these five solvents are those best suited for the extraction of TAGs.

**Table 2:** Economic and environmental studies allow one to have an idea of better solvents for microbial extraction in industry.

Solvents	Boiling Point (°C)	Toxicity Indication	Energy to Evaporate 1 kg of Solvent (kW·h)	% E Compared to Hexane	Masse CO_2_ (g) Generated by kg of Solvent	% m Compared to Hexane	Log P
Hexane	68.5	6	0.121	Ref	96	Ref	3.94
DMC	90.5	5	0.194	160	155	161	0.15
Ethyl Acetate	73.9	5	0.127	105	102	106	0.71
Ethyl Lactate	154.5	5	0.014	12	148	154	−0.19
p-Cymene	173.9	5	0.154	127	123	128	4.02
*d*-Limonene	175.4	5	0.157	130	126	131	4.45
α-Pinene	157.9	4	0.144	119	115	120	4.37
MeTHF	79.9	4	0.126	104	101	105	0.82
CPME	105.3	4	0.132	109	106	110	1.41
IPA	73	5	0.219	181	175	182	0.16
Ethanol	72.6	5	0.265	219	212	221	−0.19
	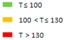	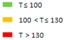		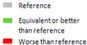		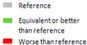	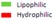

For the solubilization of DAGs and FFAs, ethyl acetate, *d*-Limonene, MeTHF and CPME are the most suitable solvents. For the phospholipids, only three solvents showed a high probability of solubilization, with RED < 1.

This software is based on only three parameters: hydrogen bonds, van der Waals forces and dipolar interactions. For a more extensive and more representative theoretical study, other parameters have to be taken into consideration. The COSMO-RS software can integrate more parameters than Hansen. This software is a tool to aid decisions and needs an experimental study.

#### 2.2.2. COSMO-RS Calculations

COSMO-RS was used to evaluate the relative solubility of the main lipid contents in the yeast *Yarrowia lipolytica* IFP29 in the selected bio-solvents. The software is based on the quantum phenomenon of target compounds shown in Figure 5. The relative solubilities of the compounds in different solvents are given by quantum calculations. The value of the best solubility is set at zero, and all solvents are given relative to the best solvent. In Table S2 in the Supplementary Materials, we see that hexane is the solvent of choice to extract TAGs compared to the other solvents tested. However, ethyl acetate, p-cymene, *d*-Limonene, α-pinene, MeTHF and CPME can also be considered as suitable solvents because of their values at zero. This means that the relative solubilities of these six bio-solvents are similar to hexane for the solubilization of TAGs of oil from *Yarrowia lipolytica*. IPA and ethanol have a low relative solubility; these results can be explained by the high polarities of IPA and ethanol compared to the other solvents.

Hexane with ethyl lactate, p-cymene, *d*-Limonene, α-pinene and ethanol are the least suitable solvents for the solubilization of DAGs. Only three solvents (ethyl acetate, MeTHF and CPME) had a high probability of solvation (value at zero).

Concerning FFAs, the four solvents with low relative solvation were hexane, p-cymene, *d*-Limonene and α-pinene. The remaining solvents can be considered better than the others for the extraction of FFAs.

Regarding phospholipid families, hexane, p-cymene, *d*-Limonene and α-pinene had a low relative solubility compared to other solvents (value included between −2 and −5). This is explained by the fact that they are non-polar solvents. DMC and ethyl lactate are bad solvents for extracting phosphatidylethanolamine, but good for solubilizing phosphatidylcholine. Conversely, CPME has values between −0.93 and −1.56; this solubility gap is due to the difference in chain length: C18:1 and C16.

According to this theoretical analysis, the choice of solvent depends on different parameters, such as the structure or polarity of the target compound. We note that hexane is not the best solvent to extract all lipids, but only TAGs. However, ethyl acetate and MeTHF are the best candidate solvents to extract all of the lipids of *Yarrowia lipolytica* IF29 (triglycerides, diglycerides, free fatty acids and phospholipid families) and are derived from renewable resources.

### 2.3. Comparison of Experimental and Theoretical Approaches

The experimental data were compared to the theoretical results. The experimental results show that the solvents had no influence on the extraction yields. However, it can be seen that the effects were significant on the distribution of lipid classes; phospholipid contents differed according to the solvent.

As we see in Table 1 and table in Supplementary Materials, the COSMO-RS theory confirms the experimental part. The simulation of different fatty acids, such as C16, C18:1n9 or C18:2n6 present on the glycerol channels, shows no influence on the behavior of the solvent.

Regarding the lipid classes with COSMO-RS and GC analysis, only hexane, p-cymene, α-pinene, *d*-Limonene, DMC and CPME extract little or no phospholipids due to their polarities. However, in the experimental part, the quantity of phosphatidylethanolamine (PE) extracted with ethyl lactate was significant, whereas the statistical study shows that it is the least suitable candidate to extract this compound. Surprisingly, phosphatidylcholine (PC) was extracted by only two solvents: isopropanol and ethanol. This can be explained by its structural difference compared to PE, as shown in Figure 5.

Some differences between theory and experiment are due to the complex composition of yeast oil. Interactions occur during extraction and can sometimes have a significant impact on the yields or lipid compositions.

COSMO-RS and HSP are prediction tools and aid decision based on many parameters. Some parameters of target compounds are unknown, such as the enthalpy of vaporization or the melting point. This study shows that we can rely on the theory, but it is necessary to complete the study by an experimental part. The theoretical and experimental studies are complementary.

### 2.4. Economic and Energy Approaches

Various parameters other than solubility have to be considered for solvent extraction of lipids from microorganisms. The boiling point, Log_10_ P, toxicity category and energy required for the evaporation of the solvent are very important parameters for the choice of the extraction solvent in industry [10]. These different parameters are shown in Table 2. For industrial use, the solvent should have a boiling point lower than 130 °C. Ethyl lactate, p-cymene, *d*-Limonene and α-pinene are solvents that are not suitable for the extraction of microbial oil in industry. Comparing the energy required to evaporate 1 kg of solvent, ethyl lactate, p-cymene, *d*-Limonene and α-pinene consume less energy than IPA, ethanol and DMC. However, we note that ethyl acetate, MeTHF and CPME have boiling points higher than hexane, but can be used industrially. These solvents consume only 5%–10% more energy and produce 5%–10% more carbon dioxide than hexane. Furthermore, these three solvents are lipophilic, with Log P > 0.

## 3. Materials and Methods

### 3.1. Strain, Culture and Harvesting Conditions

The strain of oleaginous yeast *Yarrowia lipolytica* IFP29 (ATCC 20460) was grown during 16 h in a shake flask containing 100 mL of YEPD Broth using a shaking incubator at 200 rpm, 28 °C.

The fermentation was then carried out in a 3.6-L fermenter (Labfors 4, Infors-HT) stirred with two Rushton Impellers. The oleaginous microorganism was grown in batch mode in 1 L of an industrial substrate containing hydrolyzed starch diluted to an initial concentration in glucose of 60 g/L at 28 °C and adjusted to pH 4.5. After consumption of the glucose, the fed-batch mode was started with a solution of dextrose syrup (Sirodex, Tereos Syral) and a solution of NH_4_OH as the nitrogen source.

The industrial substrate (by-product from a starch plant) was obtained from Tereos Syral. The hydrolysis of starch to glucose was carried out during 24 h at 55 °C and pH 4.5 using a commercial amyloglucosidase (Spirizyme Fuel HS, Novozymes) at a concentration of 120 µL/L.

After 72 h of fermentation, the biomass was harvested by centrifugation at 5000 rpm for 15 min and washed once with demineralized water to remove the residual nutrients. The biomass was freeze dried, and the resulting powder was stored in an air-tight container at −20 °C prior to use.

### 3.2. Reagents

Hexane, ethanol, MeTHF, DMC, IPA, ethyl acetate, ethyl lactate, p-cymene, α-pinene and *d*-Limonene, all technical grade, were sourced from VWR International (Darmstadt, Germany). CPME was sourced from Sigma Aldrich (Sigma Co., St. Louis, MO, USA). Methanol, chloroform, methyl acetate, diethyl ether, n-hexane, potassium chloride and water were of analytical grade and were sourced from VWR International (Darmstadt, Germany). Primuline and acetone were of analytical grade and sourced from Sigma Aldrich.

### 3.3. Lipid Extractions

**Figure 6:**
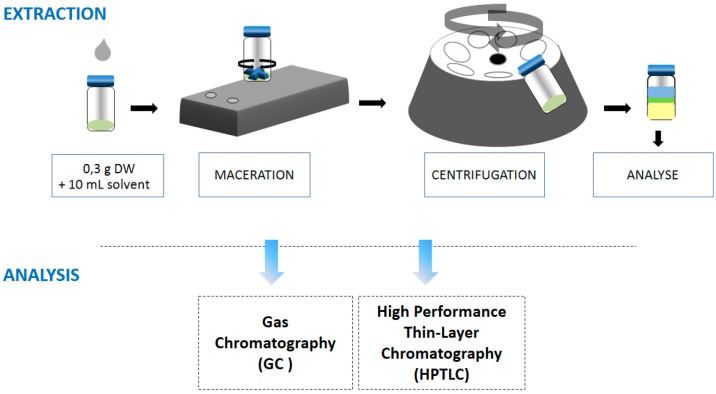
Global procedure; extractions and analysis of lipids from *Yarrowia lipolytica*.

For these experiments (Figure 6), dried yeast samples were ground before extraction to promote solubilization of lipids in each solvent. Approximately 300 mg of yeast were stirred with 15 mL each of these solvents, hexane, MeTHF, CPME, DMC, ethyl acetate, ethyl lactate, p-cymene, α-pinene, *d*-Limonene and ethanol, for 1 h. Thereafter, each sample was centrifuged (5000 rpm, 10 min, 25 °C), to separate the cellular debris.

After extraction and separation of the debris, the solvents having a lower boiling point above 100 °C, such as hexane, isopropanol, ethanol, ethyl acetate, MeTHF and CPME, were evaporated under reduced pressure.

Other solvents, such as p-cymene, *d*-Limonene and α-pinene, possessing a boiling point around 150 °C, are evaporated another way. The conventional method of evaporation under pressure is not possible for these solvents with high boiling. Moreover, terpenes are the main constituents of essential oils from plants and flowers, which are extracted by azeotropic distillation with water [11]. The addition of 50% (*v*/*v*) of water in the extraction solvent lowers its boiling point. To eliminate ethyl lactate, it is necessary to add 70% of water [12]. The several mixtures are evaporated under reduced pressure as described previously.

After evaporation, all extracts are taken up in a mixture of methanol/chloroform (1/2, *v*/*v*). Lipid yields and relative composition in fatty acids are obtained by gas chromatography and lipid classes by high performance thin-layer chromatography.

### 3.4. Qualitative and Quantitative Analysis of Lipids

#### 3.4.1. High Performance Thin-Layer Chromatography

Lipid classes were determined by two different developments chromatography to separate polar and neutral classes. Lipids were quantified by a CAMAG 3 TLC scanning densitometer (CAMAG, Muttenz, Switzerland) with identification of the classes against known polar and neutral lipid standards. Lipid extracts were loaded as a spot onto 20 × 10 cm Silica gel 60 F254 HPTLC plates (Merck KGaA, Darmstadt, Germany) using an ATS 5 automatic TLC sampler (CAMAG) The HPTLC silica gel plates were developed with a mixture of solvents in an ADC2 automatic developing chamber (CAMAG). The first eluent to separate polar lipids was a mixture of methyl acetate/isopropanol/chloroform/methanol/KCl (0.25% solution) in a ratio of 25:25:25:10:9 *v*/*v*/*v*/*v*/*v* running to a height of 7 cm from the origin. Additionally, the second eluent was a mixture of *n*-hexane/diethyl ether/glacial acetic acid in a ratio of 70:30:2 *v*/*v*/*v* to a height of 7 cm from the origin. After being dried, the plate was dipped for 6 seconds in a reagent (10 mg of primuline, 160 mL of acetone, 40 mL of water), then scanned using a TLC Scanner 3 with WinCATs software (CAMAG). Lipid classes were identified and quantified against those of corresponding lipid standards.

#### 3.4.2. Analysis by Gas Chromatography

##### Preparation of Fatty Acids Methyl Esters (FAMEs)

FAMEs were prepared from the lipid extract using acid-catalyzed transmethylation as described by Li *et al*. [13]. One milliliter of methanolic sulfuric acid (5%) solution was added to a specific amount of extracted oil. Triheptadecanoin (C17:0 TAG) was used as the internal standard. The mixture was then heated during 90 min at 85 °C. After, the flask was removed from heat, and 1.5 mL of sodium chloride (0.9%) solution and 1 mL of n-hexane were added. The flask was closed and shook vigorously during 30 s. A small amount of the organic layer was recovered and transferred to a vial before being injected directly in GC-FID for analysis.

##### FAMEs Analysis

Fatty acid methyl esters were separated, identified and quantified by gas chromatography coupled with a flame ionization detector (GC/FID). Analyses were performed by using an Agilent (Kyoto, Japan) gas chromatograph. The instrument was equipped with a BD-EN14103 capillary column 30 m × 320 μm × 0.25 μm (Agilent), and the velocity of the carrier gas (He) was at 33 cm·s^−1^. Injection of 2 μL of the various samples was carried out with a split mode (split ratio 1:20), and the injector temperature was set at 250 °C. The oven temperature was initially 50 °C for 1 min and then progressed at a rate of 20 °C/min from 50 °C–180 °C and then increased from 180 °C–220 °C at a rate of 2 °C/min. The temperature was then held at 230 °C over 10 min. FAMEs in each extract were identified by retention time and comparison with purified FAME standards (Sigma Co.).

### 3.5. Computational Method: Theoretical Prediction

#### 3.5.1. Hansen Solubility Parameters

**Figure 7:**
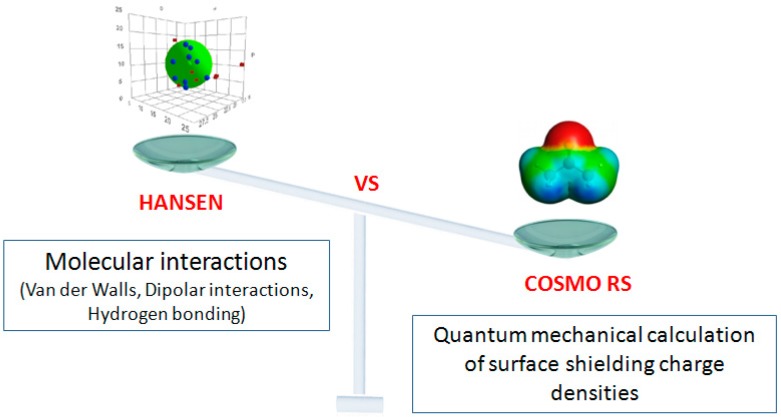
Comparison of computational methods: Hansen *vs*. COSMO-RS.

Hansen solubility parameters (HSPs) (Figure 7) provide a convenient and efficient way to characterize solute-solvent interactions according to the classical “like dissolves like” rule. HSP is based on the concept that the total cohesive energy density is approximated by the sum of the energy densities required to overcome atomic dispersion forces (δd^2^), molecular polar forces arising from dipole moments (δp^2^) and hydrogen bonds (exchange of electrons, proton donor/acceptor) between molecules (δh^2^), as given by:

δtotal^2^ = δd^2^ + δp^2^ + δh^2^ (1)

where δtotal is the Hansen total solubility parameter, which now consists of three Hansen solubility parameters for dispersion (δd), polar (δp) and hydrogen bonding (δh).

The chemical structures of the solvents and solutes discussed here were transformed by JChemPaint Version 3.3 (GitHub Pages, San Francisco, CA, USA) to their simplified molecular input line entry syntax (SMILES) notations, which were then used to calculate the solubility parameters of various alternative solvents and constituents in extracted microbial oil. Using HSP software, the relative energy difference (RED) (Equation (2)) number can be calculated to determine whether the alternative solvent and the solute are miscible:

RED = Ra/Rb
(2)

where Rb is the radius of a Hansen solubility sphere and Ra is the distance of a solvent located inside the Hansen solubility sphere.

According to the classical “like to like” rule, the smaller Ra, the greater the affinity between solute and solvent [11]. This means that a potentially good solvent has an RED number smaller than 1, while unsuitable solvents have an RED number greater than 1. These solubility parameters were further modelled to a frequently-used two-dimensional HSP sphere for better visualization of the solute/solvent interaction, because of insignificant differences between δd (HSPiP Version 4.0, Hansen-Solubility, Horsholm, Denmark).

#### 3.5.2. COSMO-RS Procedure

The conductor-like screening model for real solvents (COSMO-RS) [14] is known as a powerful method for molecular description and solvent screening based on a quantum chemical approach. COSMO-RS combines quantum chemical considerations (COSMO) and statistical thermodynamics (RS) to determine and predict thermodynamic properties without experimental data.

**Figure 8:**
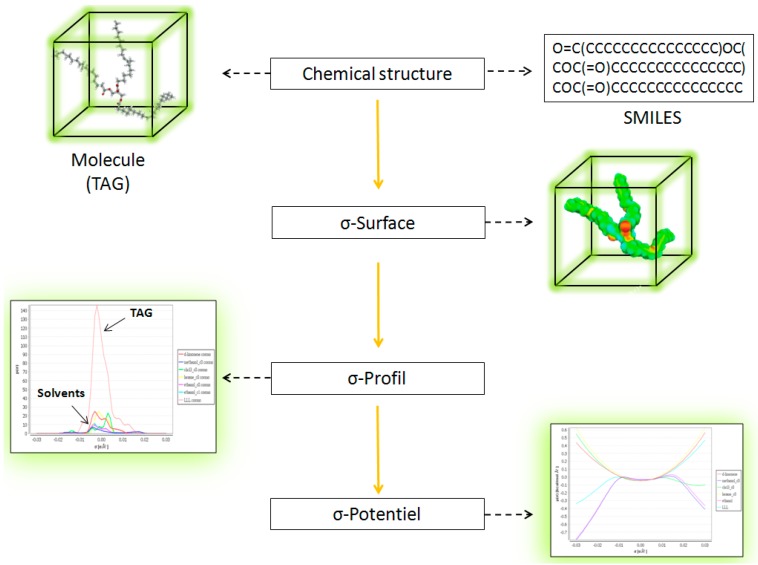
COSMO-RS procedure. SMILES, simplified molecular input line entry syntax.

In COSMO calculations, the molecules are considered in a virtual conductor environment. In such an environment, a solute molecule induces a polarization charge density on the interface between the molecule and the conductor on the molecular surface. These charges act back on the solute and generate a more polarized electron density than in a vacuum. Calculation was performed for each molecule of interest (Figure 4). The polarization charge density σ is a good local descriptor of the molecular surface polarity. The (3D) polarization density distribution on the surface of each molecule is converted into a distribution function, the “σ-profile”, which gives the relative amount of surface with polarity σ on the surface of the molecule. Blue is used to represent strongly positive polar regions, and red represents very negative polar surfaces. Green and yellow correspond to lower polarity (Figure 8) [15].

To obtain the chemical potential of the σ-surface, thermodynamic calculations of the molecular interactions based on the σ-profile obtained were carried out. The resulting chemical potentials are based on other thermodynamic equilibrium properties, such as activity coefficients, solubility, partition coefficients, vapor pressure and free energy of solvation, as well as on the σ-profile of the solvent and chemical potential. The σ-potential measures the affinity of the solvent for the solute [16,17].

**Figure 9:**
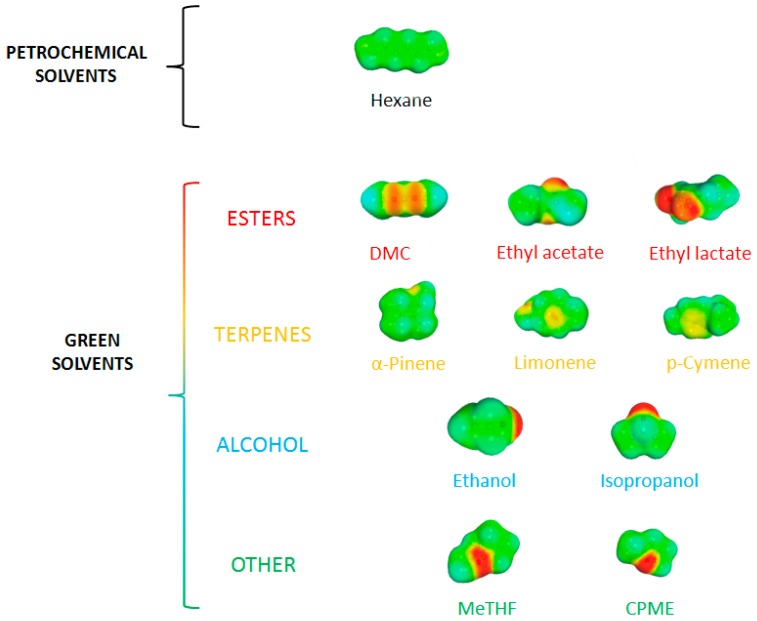
Modelization of solvents by COSMO-RS show the polarity in the surface of solvents.

For this work, the model is based on the prediction of the chemical potential of a substance in the liquid solvent [18]. Calculations of the relative solubility of typical triglycerides (Figure 9) (TAGs), diglycerides (DAGs), monoglycerides (MAGs), fatty acids (FFAs) and phospholipids (PLs) of microbial oil in various solvents were done by implementing this COSMO-RS model on COSMOtherm software (C301401, CosmothermX14, COSMOlogic GmbH & Co. KG, Cosmologic, Leverkusen, Germany). Relativesolubility is calculated from (COSMOlogic GmbH & Co. KG, 2013, Cosmologic, Leverkusen, Germany):

(3) $$\log_{10}(x_{j})=\log_{10}\left[\frac{\exp\left(\mu_{j}^{pure}-\mu_{j}^{solvent}-\Delta G_{j,fusion}\right)}{RT}\right]$$

$\mu_{j}^{pure}$: chemical potential of pure compound *j* (Joule/mol). $\mu_{j}^{solvent}$ chemical potential of *j* at infinite dilution (Joule/mol). ∆*G_j,fusion_*: free energy of fusion of *j* (Joule/mol). *x_j_*: solubility of *j*. (g/g solvent).

Relative solubility is always calculated in infinite dilution. The logarithm of the best solubility is set to 0, and all other solvents are given relatively to the best solvent. A solvent with a log_10_($x_{j}$) value of −1.00 yields a solubility that decreased by a factor 10 compared to the best solvents.

## 4. Conclusions

The present work has reported that bio-based solvents could be an alternative to petrochemical solvents, such as hexane. Some differences are noted between experimental and theoretical studies, which can be explained by the complex composition of oil from Yarrowia lipolytica IFP29.

Some differences are noted between experimental and theoretical studies that can be explained by the complex composition of oil from *Yarrowia lipolytica* IFP29.

Ethyl acetate, CPME and MeTHF could replace hexane for the solubilization of microbial oils. Economically and technically, these solvents have properties similar to those of hexane and more specifically for the energy required to evaporate 1 kg of solvent and carbon footprint. By contrast, theoretical and economic studies show that *d*-Limonene, p-cymene, α-pinene, ethyl lactate, IPA and ethanol are not suitable for replacing hexane, even for the solubility of oil, classes of lipids or for technical and economic reasons.

## Supplementary Materials

Supplementary materials can be accessed at: http://www.mdpi.com/1420-3049/21/2/196/s1.

## Abbreviations

The following abbreviations are used in this manuscript:

COSMO-RS: conductor-like screening model for realistic solvation
HSP: Hansen solubility parameters
RED: relative energy difference
HPTLC: high performance thin-layer chromatography
GC: gas chromatography
FID: flame ionization detector
CPME: cyclopentyl methyl ether
MeTHF: 2-methyltetrahydrofuran
DMC: dimethyl carbonate
IPA: isopropanol
EtOAc: ethyl acetate
TAGs: triglycerides
DAGs: diglycerides
MAGs: monoglycerides
FFAs: free fatty acids
PLs: phospholipids
FAMEs: fatty acid methyl esters
GRAS: generally recognized as safe
DOAJ: Directory of Open Access Journals
TLA: three letter acronym
LD: linear dichroism

## References

[1] Tanzi C.D., Vian M.A., Chemat F. (2013). New procedure for extraction of algal lipids from wet biomass: A green clean and scalable process. Bioresour. Technol..

[2] Cescut J. (2009). Accumulation D’acylglycérols par des Espèces Levuriennes à Usage Carburant Aéronautique: Physiologie et Performances de Procédés. Ph.D. Thesis.

[3] Ratledge C. (1951). Microorganisms for lipids. Acta Biotechnol..

[4] Klug M.J., Markovetz A.J. (1967). Degradation of Hydrocarbons by Members of the Genus Candida II. Oxidation of n-Alkanes and 1-Alkenes by Candida lipolytica. J. Bacterial..

[5] Lodder J., Kreger-van Rij N.J.W. (1952). The Yeasts-A Taxonomic Study.

[6] Vian M.A., Tanzi C.D., Chemat F. (2013). Techniques conventionelles et innovantes, et solvants alternatifs pour l’extraction des lipides de micro-organismes. OCL.

[7] Battershill J.M., Illing H.P.A., Shillaker R.O., Smith A.M. (1987). *n*-Hexane. Health & Safety Executive Toxicity Review.

[8] Chemat F. (2014). Alternative Solvents for Natural Products Extraction. Ph.D. Thesis.

[9] Fine F., Vian M.A., Tixier A.S.F., Carre P., Pages X., Chemat F. (2013). Les agro-solvants pour l’extraction des huiles végétales issues de graines oléagineuses. OCL.

[10] Sicaire A.G., Vian M.A., Fine F., Carré P., Tostain S., Chemat F. (2015). Experimental approach *versus* COSMO-RS assisted solvent screening for predicting the solubility of rapeseed oil. OCL.

[11] Filly A., Fabiano-Tixier A.S., Lemasson Y., Roy C., Fernandez X., Chemat F. (2014). Extraction of aroma compounds in blackcurrant buds by alternative solvents: Theoretical and experimental solubility study. Comptes Rendus Chim..

[12] Sicaire A.G., Vian M., Fine F., Joffre F., Carré P., Tostain S., Chemat F. (2015). Alternative Bio-Based Solvents for Extraction of Fat and Oils: Solubility Prediction, Global Yield, Extraction Kinetics, Chemical Composition and Cost of Manufacturing. Int. J. Mol. Sci..

[13] Morrison W.R., Smith L.M. (1964). Preparation of fatty acid methyl esters and dimethylacetals from lipids with boron fluoride–methanol. J. Lipid Res..

[14] COSMOlogic GmbH & Co. KG (2013). COSMOthermpX Tutorial.

[15] Klamt A., Eckert F., Arlt W. (2010). COSMO-RS: An alternative to simulation for calculating thermodynamic properties of liquid mixtures. Annu. Rev. Chem. Biomol. Eng..

[16] Klamt A., Krooshof G.J., Taylor R. (2002). COSMOSPACE: Alternative to conventional activity-coefficient models. AIChE J..

[17] Filly A., Fabiano-Tixier A.S., Fernandez X., Chemat F. (2015). Alternative solvents for extraction of food aromas. Experimental and COSMO-RS study. LWT-Food Sci. Technol..

[18] Mehler C., Klamt A., Peukert W. (2002). Use of COSMO-RS for the prediction of adsorption equilibria. AIChE J..

