# Supplementary material for: Bio-Based Solvents for Green Extraction of Lipids from Oleaginous Yeast Biomass for Sustainable Aviation Biofuel
Source: Molecules. 2016 Feb 6;21(2):196. doi: 10.3390/molecules21020196 (PMC6274296; doi:10.3390/molecules21020196)
Supplement: Supplementary file 1 [file molecules-21-00196-s001.pdf]

Supplementary Materials: Bio-Based Solvents for Green Extraction of Lipids from Oleaginous Yeast Biomass for Sustainable Aviation Biofuel

Cassandra Breil, Alice Meullemiestre, Maryline Vian and Farid Chemat

Table S1. The aim is to see the difference of the solubilization of major molecules present in yeast with different solvents. The relative energy difference (RED) values of agro-solvents for the extraction allows one to have the prediction of the solubilization of major compounds.

Solvents	∂D	∂P	∂H	TAG 1	TAG 2	TAG 3	TAG 4	TAG 5	TAG 6	TAG 7	DAG 1	DAG 2	DAG 3	FFA 1	FFA 2	FFA 3	PE 1	PE 2	PE 3	PE 4	PC 1	PC 2	PC 3	PC 4
Hexane	14	0	0	1.1	1.14	1.13	1.17	1.11	1.07	1.1	1.85	1.84	1.89	1.86	1.82	2	3.05	2.97	2.89	2.93	2.74	2.72	2.7	2.73
DMC	15.5	8.6	9.7	2.61	2.55	2.51	2.46	2.53	2.58	2.48	1.61	1.64	1.67	1.64	1.75	1.72	0.69	0.68	0.71	0.64	1.06	0.97	0.89	0.84
Ethyl Acetate	15.8	5.3	7.2	1.59	1.53	1.48	1.43	1.51	1.56	1.46	0.58	0.6	0.64	0.61	0.73	0.75	0.8	0.71	0.63	0.66	0.66	0.57	0.49	0.5
Ethyl Lactate	16	7.6	12.5	2.97	2.92	2.87	2.82	2.9	2.96	2.85	1.9	1.92	1.92	1.94	2.04	1.95	0.73	0.8	0.88	0.83	1.05	1.04	1.04	1
p-Cymene	17.3	2.3	2.4	0.39	0.38	0.43	0.44	0.42	0.43	0.47	1.19	1.15	1.11	1.07	0.94	1.01	2.2	2.13	2.06	2.11	1.05	1.93	1.9	1.95
Limonene	16.7	1.8	3.1	0.59	0.54	0.53	0.49	0.57	0.61	0.57	0.86	0.81	0.75	0.74	0.61	0.64	1.87	1.81	1.74	1.8	1.58	1.57	1.56	1.61
a-Pinene	16.4	1.3	2.2	0.13	0.16	0.17	0.22	0.15	0.11	0.18	1.15	1.12	1.12	1.09	1	1.14	2.32	2.25	2.17	2.22	2.03	2	1.99	2.03
MeTHF	16.4	4.7	4.6	1	0.95	0.94	0.9	0.94	0.99	0.93	0.81	0.77	0.75	0.66	0.6	0.64	1.47	1.4	1.33	1.37	1.37	1.3	1.23	1.27
CPME	16.7	4.3	4.3	0.84	0.79	0.77	0.72	0.78	0.83	0.76	0.67	0.63	0.62	0.52	0.46	0.54	1.51	1.44	1.36	1.41	1.35	1.29	1.23	1.27
IPA	15.8	6.1	16.4	3.72	3.67	3.61	3.56	3.65	3.71	3.6	2.63	2.66	2.65	2.7	2.79	2.69	1.66	1.72	1.79	1.76	1.75	1.81	1.87	1.84
Ethanol	15.8	8.8	19.4	4.66	4.6	4.55	4.49	4.59	4.64	4.54	3.56	3.59	3.58	3.62	3.72	3.61	2.46	2.53	2.61	2.57	2.67	2.71	2.74	2.71

RED > 1 RED < 1

Triglycerides: TAG 1 (R1: C18:2n-6, R2: C18:2n6, R3: C16), TAG 2 (R1: C18:2n-6, R2: C18:2n6, R3: C18:2n-6), TAG 3 (R1: C18:2n-6, R2: C18:1n9, R3: C16), TAG 4 (R1: C18:1n9, R2: C18:1n9, R3: C18:1n9), TAG 5 (R1: C18:1n9, R2: C18:2n-6, R3: C18:2n6), TAG 6 (R1: C18:1n-9, R2: C18:1n9, R3: C16), TAG 7 (R1: C18:2n6, R2: C18:1n-9, R3: C18:1n9).

Diglycerides: DAG 1 (R1: C18:2n6, R2: C18:2n6), DAG 2 (R1: C18:2n6, R2: C18:1n9), DAG 3 (R1: C18:1n9, R2: C18:1n9).

Free Fatty Acids: FFA 1 (R1: C16), FFA 2 (R1: C18:2n6), FFA 3 (R1: C18:2n6).

Phosphatidylethanolamine: PE 1 (R1: C18:2n-6, R2: C18:2n6), PE 2 (R1: C18:1n9, R2: C18:2n-6), PE 3 (R1: C18:1n9, R2: C18:1n9), PE 4 (R1: C16, R2: C18:1n9).

Phosphatidylcholine: PC 1 (R1: C18:2n-6, R2: C18:2n6), PC 2 (R1: C18:1n9, R2: C18:2n-6), PC 3 (R1: C18:1n9, R2: C18:1n9), PC 4 (R1: C16, R2: C18:1n9).

Table S2. The aim is to show the difference of the solubilization of major molecules present in yeast with different solvents. COSMO–RS prediction results regarding the solubility of the major components of Yarrowia lipolytica oil in several solvents.

Solvent	TAG 1	TAG 2	TAG 3	TAG 4	TAG 5	TAG 6	TAG 7	DAG 1	DAG 2	DAG 3	FFA 1	FFA 2	FFA 3	PE 1	PE 2	PE 3	PE 4	PC 1	PC 2	PC 3	PC 4
Hexane	0	−0.03	−0.02	0	0	0	0	−1.66	−1.46	−1.18	−1.29	−1.36	−1.33	−5.27	−4.46	−4.03	−4.51	−5.31	−6.52	−4.53	−5.23
DMC	−1.46	−1.71	−1.36	−1.46	−1.62	−2.28	−1.42	−0.34	−0.38	−0.34	−0.53	−0.49	−0.19	−1.17	−0.79	−1.58	−1.28	0	−0.01	0	0
Ethyl Acetate	0	0	0	0	0	−0.24	0	0	0	0	0	0	0	0	0	0	−0.19	0	0	0	0
Ethyl Lactate	−3.26	−2.74	−3.15	−3.23	−3.52	−3.99	−3.24	−1.07	−1.87	−1.53	−0.22	−0.27	−0.06	−0.63	−0.56	−1.04	−1.08	0	0	0	0
p-Cymene	0	0	0	0	0	0	0	−0.83	−0.88	−0.74	−1.03	−1.03	−0.91	−3.65	−2.92	−3.05	−3.35	−2.51	−3.41	−2.1	−2.6
Limonene	0	0	0	0	0	0	0	−0.88	−0.73	−0.79	−0.96	−0.98	−0.89	−3.81	−3.09	−3.07	−3.4	−3.01	−3.97	−2.49	−3.05
α-Pinene	0	0	0	0	0	0	0	−1.11	−0.96	−0.86	−1.08	−1.11	−1.03	−4.24	−3.48	−3.39	−3.75	−3.54	−4.55	−2.98	−3.56
MeTHF	0	0	0	0	0	0	0	0	0	0	0	0	0	0	0	0	0	0	0	0	0
CPME	0	0	0	0	0	0	0	0	0	0	0	0	0	0	0	0	0	−0.93	−1.56	−0.95	−0.97
IPA	−1.67	−1.43	−1.56	−1.52	−1.77	−2.04	−1.55	−0.24	−0.22	−0.36	0	0	0	0	0	−0.025	−0.1	0	0	0	0
Ethanol	−2.57	−2.23	−2.46	−2.46	−2.72	−3.08	−2.45	−0.72	−0.76	−0.97	0	−0.03	0	−0.19	−0.19	−0.4	−0.52	0	0	0	0

0   -0,1 ≤ x ≤ -0,99   -1 ≤ x ≤ -5

Triglycerides: TAG 1 (R1: C18:2n–6, R2: C18:2n6, R3: C16), TAG 2 (R1: C18:2n–6, R2: C18:2n6, R3: C18:2n–6), TAG 3 (R1: C18:2n–6, R2: C18:1n9, R3: C16), TAG 4 (R1: C18:1n9, R2: C18:1n9, R3: C18:1n9), TAG 5 (R1: C18:1n9, R2: C18:2n–6, R3: C18:2n6), TAG 6 (R1: C18:1n–9, R2: C18:1n9, R3: C16), TAG 7 (R1: C18:2n6, R2: C18:1n–9, R3: C18:1n9).

Diglycerides: DAG 1 (R1: C18:2n6, R2: C18:2n6), DAG 2 (R1: C18:2n6, R2: C18:1n9), DAG 3 (R1: C18:1n9, R2: C18:1n9).

Free Fatty Acids: FFA 1 (R1: C16), FFA 2 (R1: C18:2n6), FFA 3 (R1: C18:2n6).

Phosphatidylethanolamine: PE 1 (R1: C18:2n–6, R2: C18:2n6), PE 2 (R1: C18:1n9, R2: C18:2n–6), PE 3 (R1: C18:1n9, R2: C18:1n9), PE 4 (R1: C16, R2: C18:1n9).

Phosphatidylcholine: PC 1 (R1: C18:2n–6, R2: C18:2n6), PC 2 (R1: C18:1n9, R2: C18:2n–6), PC 3 (R1: C18:1n9, R2: C18:1n9), PC 4 (R1: C16, R2: C18:1n9).
